# Hypothermic circulatory arrest does not induce coagulopathy in vitro

**DOI:** 10.1007/s10047-022-01324-5

**Published:** 2022-03-18

**Authors:** Hayato Ise, Kyohei Oyama, Shingo Kunioka, Tomonori Shirasaka, Hirotsugu Kanda, Payam Akhyari, Hiroyuki Kamiya

**Affiliations:** 1 Department of Cardiac Surgery, Asahikawa Medical University, Midorigaoka-Higashi 2-1-1-1, Asahikawa, Hokkaido 078-8510 Japan; 2 Department of Cardiovascular Surgery, Heinrich Heine University, Düsseldorf, Germany; 3 Department of Anesthesiology and Critical Care Medicine, Asahikawa Medical University, Asahikawa, Japan

**Keywords:** Hypothermic circulatory arrest, Cardiopulmonary bypass, Coagulopathy, Bleeding, In vitro study

## Abstract

Hypothermic circulatory arrest (HCA) is an essential procedure during aortic surgery to protect organs; however, hypothermia is believed to cause coagulopathy, which is a major fatal complication. This study aimed to clarify the impact of hypothermia on coagulation by eliminating clinical biases in vitro. In the hypothermic storage study, blood samples from five healthy volunteers were stored at 37 ℃ (group N) for 3 h or at 20 ℃ for 2 h, followed by 1 h of rewarming at 37 ℃ (group H). Thromboelastography was performed before and after 3 h of storage. In the mock circulation loop (MCL) study, blood samples were placed in the MCL and (a) maintained at 37 ℃ for 4 h (group N, *n* = 5), or (b) cooled to 20 ℃ to simulate HCA with a 0.1 L/min flow rate for 3 h and then rewarmed to 37 ℃ (group H, *n* = 5). The total MCL duration was 4 h, and the flow rate was maintained at 1 L/min, except during HCA. Blood samples collected 15 min after the beginning and end of MCL were subjected to standard laboratory tests and rotational thromboelastometry analyses. Hypothermia had no impact on coagulation in both the hypothermic storage and MCL studies. MCL significantly decreased the platelet counts and clot elasticity in the INTEM and EXTEM assays; however, there was no effect on fibrinogen contribution measured by FIBTEM. Hypothermia does not cause irreversible coagulopathy in vitro; however, MCL decreases coagulation due to the deterioration of platelets.

## Introduction

Hypothermic circulatory arrest (HCA) is an essential procedure to protect organs during aortic surgery while performing distal anastomosis in blood-free fields [1, 2]. In 1975, Griepp et al. reported the benefits of deep hypothermic circulatory arrest (DHCA) for aortic surgery [3]. Subsequently, the use of antegrade or retrograde cerebral perfusion combined with HCA was developed [4–6], dramatically improving the outcomes and reducing the complications of aortic surgery.

Coagulopathy is a major fatal complication caused by hypothermia; therefore, an increase in temperature during HCA tends to lower the risk of coagulopathy [7]. However, a higher temperature setting during HCA does not ensure the protection of organs in cases of unanticipated prolongation of the procedure [8]. Moreover, the contention that hypothermia during circulatory arrest causes coagulopathy has not been proven in previous studies.

Thus, in this study, we aimed to clarify the influence of hypothermia on blood coagulation in vitro by eliminating other clinical factors.

## Materials and methods

### Ethical approval

The Institutional Review Board at Asahikawa Medical University approved this study (approval number: 18210 on 1/28/2019 & 19001 on 7/29/2019). Written informed consent was obtained from all volunteers who participated in the study.

### Volunteers

The participants had no medical history of hemostasis and medications. Five healthy male volunteers were recruited for the hypothermic storage study, and 10 healthy volunteers (six men and four women) were recruited for the mock circulation loop (MCL) study.

### Measurement of blood coagulation

#### Thromboelastography (TEG)

The TEG analyses were performed using TEG 6 s (Haemonetics Corp., Braintree, MA, USA) according to the manufacturer’s instructions. The analyses included TEG global hemostasis and TEG platelet mapping. For the former, kaolin was used with standard TEG (CK) to activate blood coagulation and monitor the intrinsic pathway-induced clot formation. For rapid TEG (CRT), tissue factors and kaolin were used as activators to monitor clot formation induced by the intrinsic and extrinsic pathways. Functional fibrinogen (CFF) was used to activate extrinsic pathways in the presence of GPIIb/IIIa and block platelet activation, allowing the assessment of fibrinogen effects on clot formation. In TEG platelet mapping, activator F (ActF), which includes reptilase and factor XIII, was used to produce clots without platelet contribution. Adenosine diphosphate (ADP) was used to activate platelets and measure their activity.

In the TEG tests, multiple parameters [reaction time (*R*), kinetic value (*K*), and maximum amplitude (MA)] were measured to evaluate clot formation. The *R* value indicates the time until the first evidence of clot formation, while the *K* value is the time from the beginning of clot formation to it reaching 20 mm, representing the speed of formation. The MA represents the maximum strength of the clot. This study analyzed CRT-*R*, CRT-*K*, CRT-MA, CK-*R*, CK-*K*, CK-MA, and CFF-MA during TEG global hemostasis, and ActF-MA and ADP-MA in TEG platelet mapping.

#### Standard laboratory tests (SLTs)

SLTs, including hemoglobin content (Hb), hematocrit (Hct), platelet count (Plt), fibrinogen concentration (Fib), antithrombin III level (AT3), prothrombin time (PT), and activated thromboplastin time (APTT), were measured using a fully automated hematology analyzer XN-3000 (Sysmex Corporation, Kobe, Japan) and a fully automated coagulation analyzer CS-5100 (Sysmex Corporation) at the central lab of Asahikawa Medical University Hospital.

#### Rotational thromboelastometry (ROTEM)

ROTEM was performed to assess clot formation properties using ROTEM delta (Tem Systems Inc., Munich, Germany) according to the manufacturer’s instructions. In this study, we used INTEM, EXTEM, and FIBTEM. The INTEM and EXTEM were used to monitor the intrinsic and extrinsic pathway-induced clot formation, respectively, using ellagic acid and tissue factor as activators of clot formation, respectively. The FIBTEM activates the extrinsic pathway using tissue factors in the presence of cytochalasin D to block the platelets and allow for fibrinogen function to be assessed.

In ROTEM analyses, clotting time (CT) is the latent time before the start of clot formation, and clot formation time (CFT) is the time from CT until the clot is 20 mm in size, indicating the speed of clot formation. The maximum clot firmness (MCF) is the largest vertical amplitude of the trace, reflecting the absolute strength of fibrin and platelet clots. The maximum clot elasticity (MCE) is calculated as follows: (MCF × 100) / (100  MCF) to accommodate Hook’s law, and represents the actual physical properties of the clot. We used CT, CFT, MCF, and MCE in the INTEM and EXTEM analyses to assess clot formation status. Platelet MCE, an indicator of platelet contribution to clot strength, was calculated as FIBTEM-MCE subtracted from EXTEM-MCE, as previously reported [9].

### Study design

#### Hypothermic storage study

**Fig. 1:**
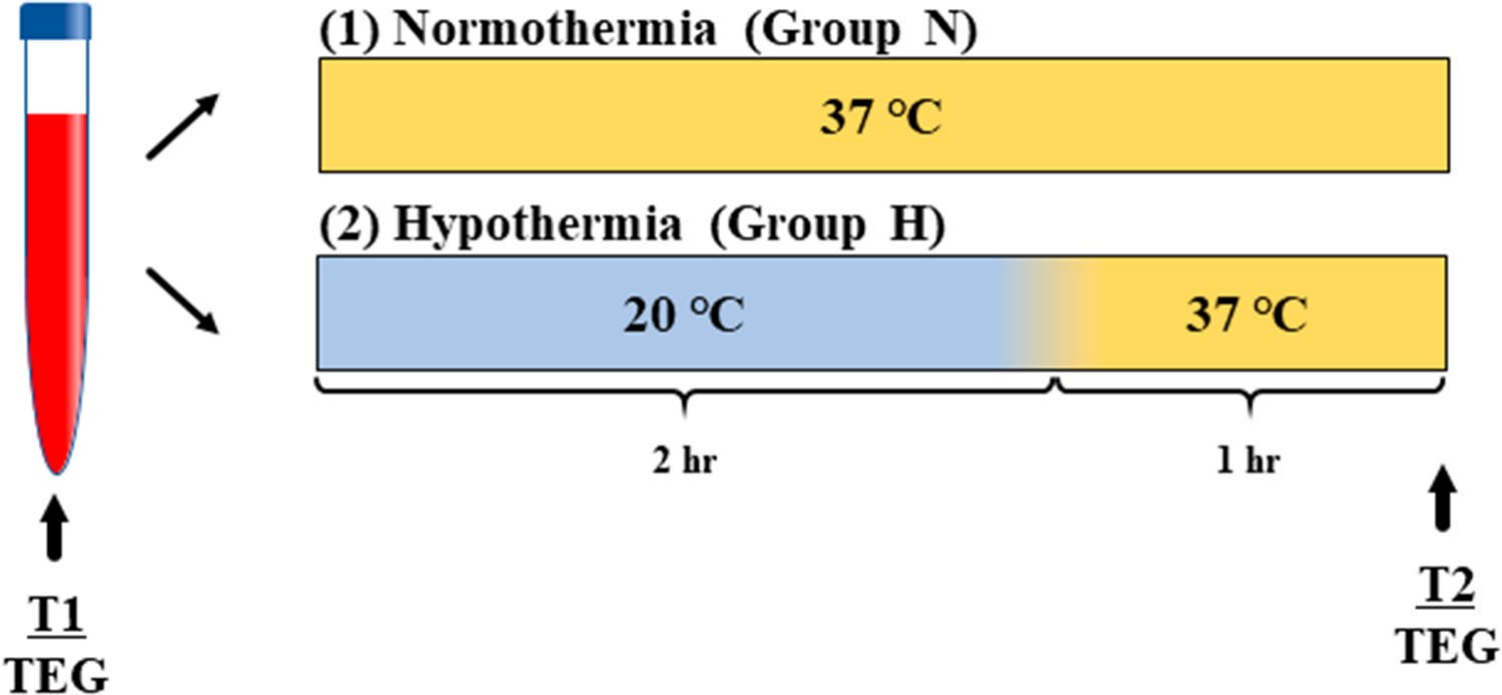
Design of the hypothermic storage study. Blood samples collected from healthy volunteers were stored at 37 °C for 3 h (group N) or at 20 °C for 2 h, followed by 1 h of rewarming (group H). Coagulation was measured before (T1) and after (T2) storage by TEG

Approximately 20 mL of venous blood was obtained from an antecubital vein and placed into three citrated vacuum tubes (2.7 mL each) (BD Vacutainer, 0.109 M sodium citrate, Cat# 364305) for the TEG global hemostasis and into three heparin vacuum tubes (2.0 mL) (BD Vacutainer, heparin lithium, Cat# 368272) for TEG platelet mapping. Half of the collected blood was incubated at 37 °C in a water bath for 3 h (normothermic condition: group N), and the other half was incubated at 20 °C for 2 h followed by rewarming at 37 °C for 1 h in a water bath (hypothermic condition: group H). The TEG tests were performed before (control; T1) and after (group N and group H; T2) 3 h of storage, and the differences in clot formation between groups N and H were compared (Fig. 1).

#### MCL study

**Fig. 2:**
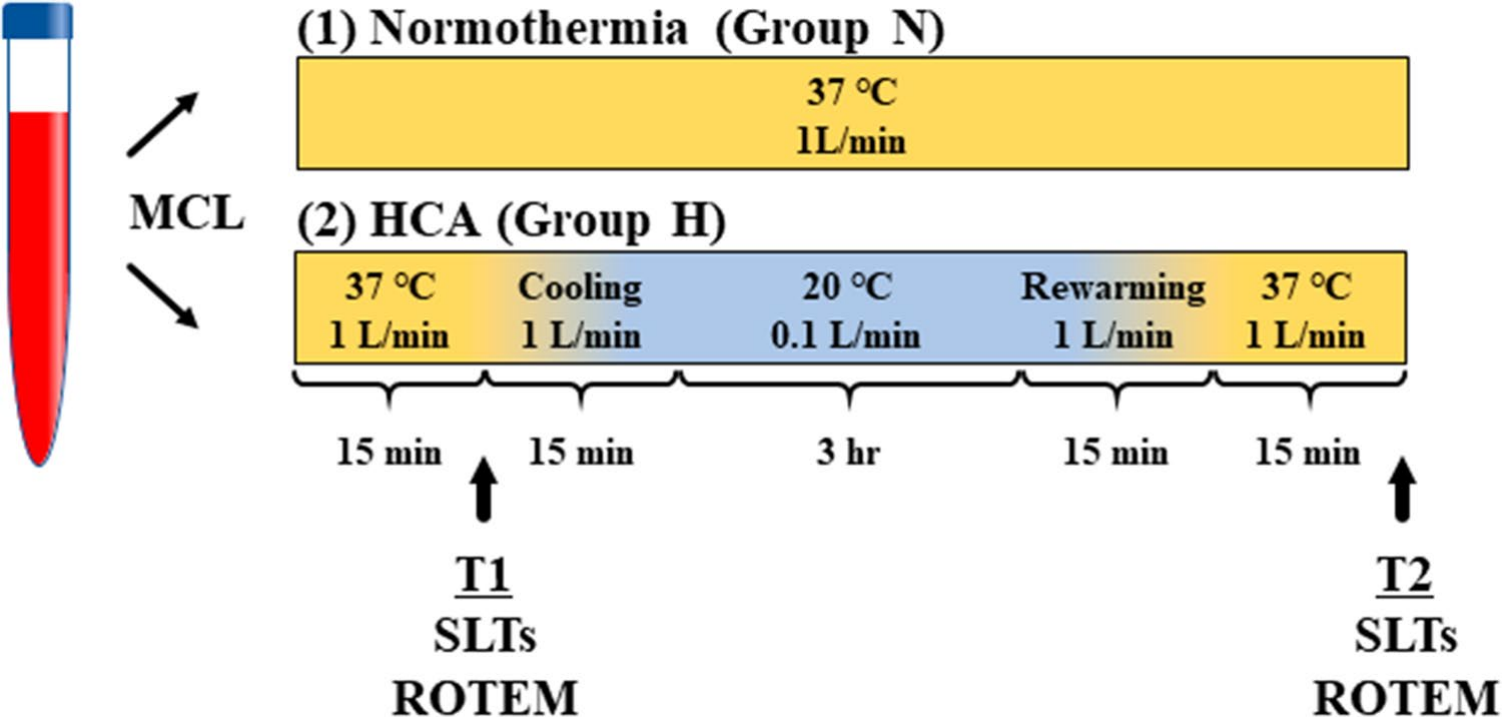
Design of the mock circulation loop (MCL) study. Blood samples were collected from healthy volunteers and applied to the MCL. The samples in group N were maintained at 37 °C and a 1 L/min flow rate for 4 h. Group H samples were maintained at 20 °C with a 0.1 L/min flow rate for 3 h, simulating HCA conditions. After 3 h, the samples were warmed for 30 min. SLTs and ROTEM analyses were performed before (T1) and after (T2) HCA simulations

Venous blood was obtained from an antecubital vein and placed into a 200 mL blood collection bag with citrate phosphate dextrose adenine (Karmi CA, Kawasumi Chemicals, Tokyo, Japan). Blood samples from volunteers were divided into groups N and H. The MCL consisted of a roller pump (Stockert S3, LivaNova, UK), reservoir, and oxygenator (CX-FX05RW, Terumo, Tokyo, Japan) with a heater unit (HHC-51, Senko Medical, Tokyo, Japan), and was primed with 150 mL of saline. Blood samples were added to the MCL within 30 min of collection. Samples in group N were maintained at 37 °C and 1 L/min flow rate during the 4-hour study. Samples in group H were placed in the MCL at 37 °C and a 1 L/min flow rate for the first 15 min, followed by 15 min of cooling till the blood temperature reached 20 °C. HCA was then simulated by maintaining the temperature at 20 °C and the flow rate at 0.1 L/min for 3 h. After HCA, the blood was rewarmed for 15 min to reach 37 °C, and the MCL was warmed back to 37 °C and a flow rate of 1 L/min for another 15 min. The total MCL time was 4 h in both groups. The blood samples were collected 15 min after the beginning (T1) and end (T2) of running the MCL for SLT and ROTEM analyses (Fig. 2).

The MCL flow rate in previous studies ranged from 0.6 to 5 L/min [10–12]. According to the manufacturer’s instructions, the speed settings of the reservoir and oxygenator used in the present study ranged from 0.1 to 1.5 L/min. Considering the available settings, we decided to use a flow rate of 1.0 L/min in group H, which is comparable to that used in previous reports and considered to be reasonable for simulating standard cardiopulmonary bypass (CPB). Conversely, we simulated HCA at the minimum flow rate of 0.1 L/min because, even when flow from the CPB circuit to the patient’s body is arrested during HCA in the clinical setting, a low flow rate is maintained through the oxygenator to prevent blood coagulation and platelet aggregation.

### Statistical analysis

**Fig. 3:**
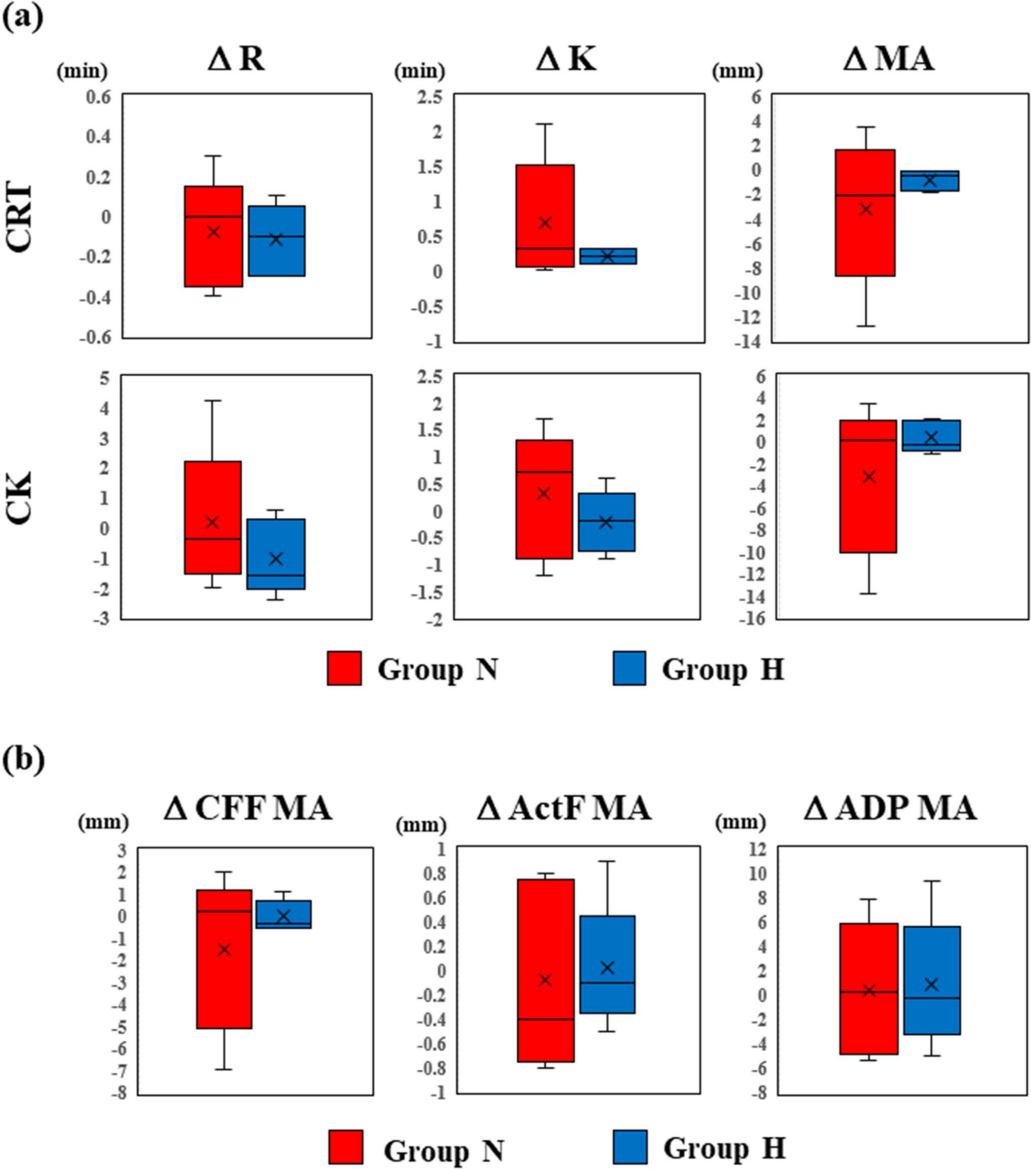
Effect of hypothermic storage on blood coagulation. Blood samples were stored at 37 °C (Group N) or 20 °C (Group H), and coagulation was measured by TEG. Shown are the changes in the resulting parameters before and after storage. **a** Intrinsic and extrinsic pathways and **b** fibrinogen and platelet contributions. The whiskers, cross, and horizontal lines in the box plots represent the maximum value (upper whisker), minimum value (lower whisker), average (cross), median of the third quartile (upper horizontal line), median of all samples (horizontal line in box), and median of the first quartile (lower horizontal line). Group N (red) and H (blue)

**Fig. 4:**
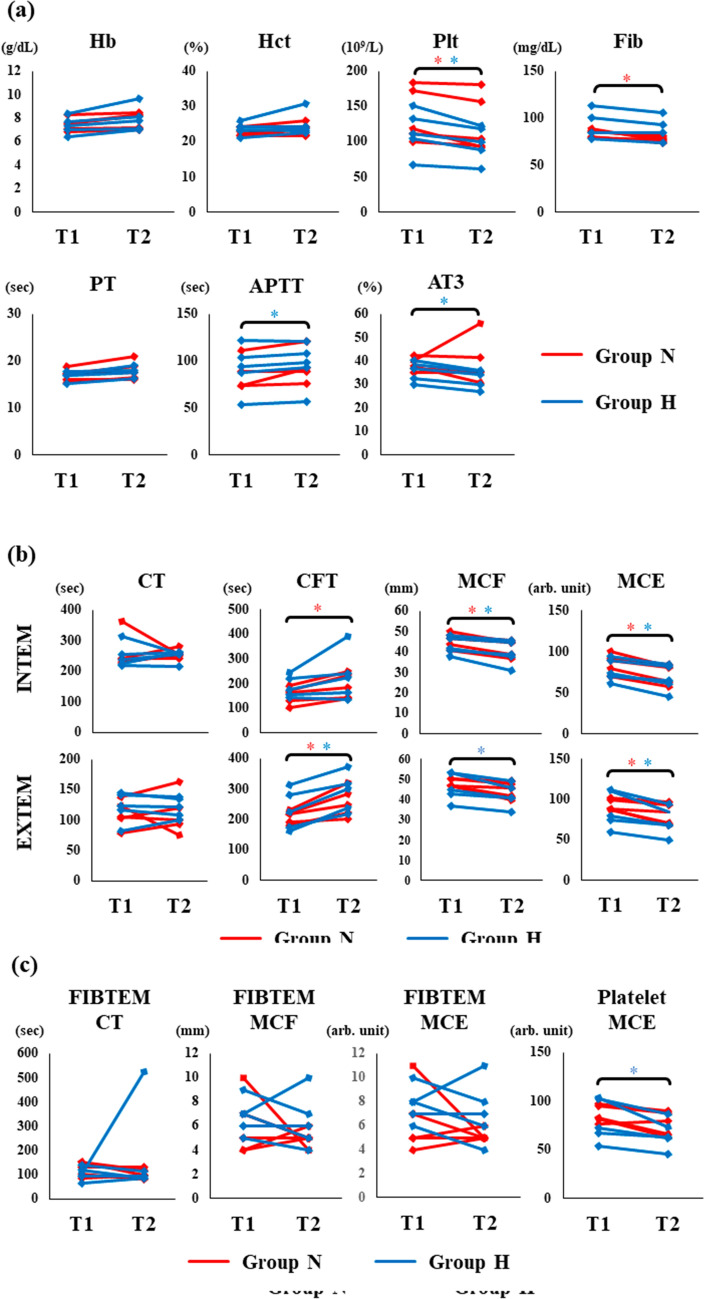
MCL reduces coagulation. Blood samples were placed into the MCL and maintained at 37 °C with a 1 L/min flow rate (Group N) or under conditions simulating HCA at 20 °C with a 0.1 L/min flow rate. SLTs and ROTEM were performed 15 min after the beginning (T1) and the end (T2) of MCL to measure coagulation. **a** SLT measurements, **b** intrinsic and extrinsic pathways, and **c** fibrinogen and platelet contributions. The data points represent the individual values at T1 and T2. Group N (red) and H (blue). * represents *p* < 0.05 (T2 vs. T1)

**Fig. 5:**
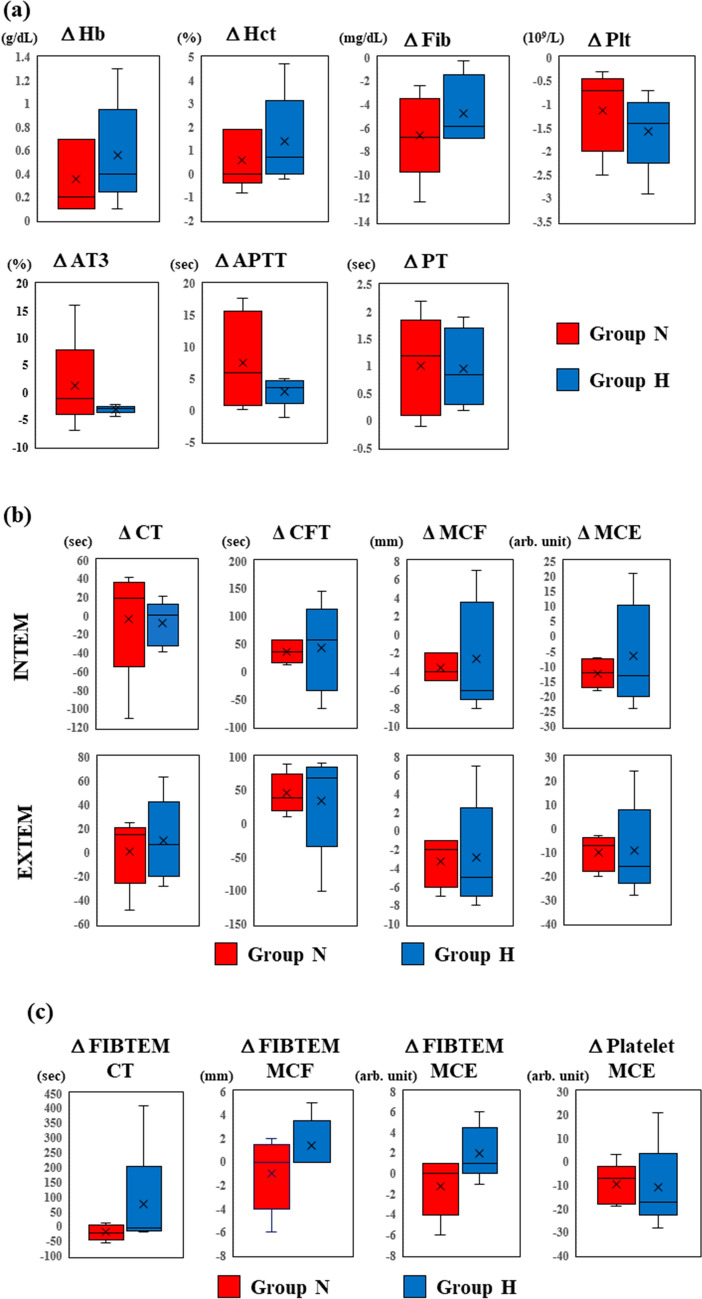
Effect of hypothermia on coagulation during MCL. Shown are the changes in parameters after MCL (T1–T2). **a** SLT measurements, **b** intrinsic and extrinsic pathways, and **c** fibrinogen and platelet contributions. The whiskers, cross, and horizontal lines in the box plots represent the maximum value (upper whisker), minimum value (lower whisker), average (cross), median of the third quartile (upper horizontal line), median of all samples (horizontal line in box), and median of the first quartile (lower horizontal line). Group N (red) and H (blue)

All statistical data were analyzed using SPSS 27.0 for Windows (IBM Corp., Armonk, NY, USA). Numerical data are described as median with quartiles (Figs. 3 and 5) or the actual measurement values (Fig. 4). Student’s t-test was used to compare variables between the H and N groups. A paired t-test was used to compare variables between the different time points in the same samples (T1 vs. T2). Statistical significance was defined as *p* < 0.05.

## Results

### Hypothermic storage study

To examine whether hypothermia causes an irreversible loss in clot formation capability, blood samples were stored at 37 °C for 3 h (group N) or 20 °C for 2 h followed by 1 h rewarming at 37 °C (group H), and clot-forming ability was measured using TEG (Fig. 1). Changes in the clotting time (*R*) and clot formation time (*K*) before and after storage were comparable between groups N and H in both the CK and CRT assays (Fig. 3a), indicating no differences in intrinsic- and extrinsic pathway-associated coagulation in the two groups.

Next, we analyzed the impact of hypothermic storage on the contribution of fibrinogen or platelets to clot strength (Fig. 3b). The two groups showed no differences in CFF-MA (global hemostasis) or ActF-MA (platelet mapping), indicating the contribution of fibrinogen to clot strength. ADP-MA, which is an indicator of the involvement of platelets in clot formation, was also comparable between the two groups. These results indicated that coagulation related to fibrinogen and platelets did not differ between the N and H groups.

Therefore, the hypothermic storage study demonstrated that exposure to hypothermic temperature did not influence coagulation in vitro.

### MCL study

Next, to investigate whether hypothermic temperature causes coagulopathy during CPB, we ran the blood through the MCL. Blood samples were collected before (T1) and after (T2) HCA simulations, and SLTs and ROTEM were performed, as shown in Fig. 2. One measurement of PT in group N and one each of APTT and FIBTEM CT in group H were excluded due to errors in the assays.

#### Effect of MCL on coagulation (T2 vs. T1)

We compared the SLT findings (Hb, Hct, Plt, Fib, PT, APTT, and AT3) at T1 and T2 in both the N and H groups (Fig. 4a). No changes were seen in Hb, Hct, and PT after MCL in either group, while a significant decrease in platelet count was seen in the N (*p* = 0.048) and H (*p* = 0.013) groups. APTT was extended (*p* = 0.047), and AT3 (*p* < 0.01) decreased significantly in group H. The fibrinogen level was decreased in group N (*p* = 0.016).

The ROTEM findings were compared to determine whether MCL affects clot formation in each group (Fig. 4b, c). In the INTEM and EXTEM assays, CT did not change after MCL in both groups. CFT was significantly extended in group N (*p* = 0.016) in the INTEM assay, and in both groups (group N: *p* = 0.029, group H: *p* < 0.01) in the EXTEM assay. The MCF was significantly decreased at T2 in both groups (group N, *p* < 0.01; group H, *p* = 0.02) in the INTEM assay, and in group H in the EXTEM assay (*p* < 0.01). MCE showed a significant decrease in both groups in the INTEM (group N, *p* < 0.01; group H, *p* < 0.01) and EXTEM (group N, *p* = 0.037; group H, *p* = 0.016) assays. However, in the FIBTEM assays, CT, MCF, and MCE did not change after MCL in either group (Fig. 4c). In contrast, the calculated platelet MCE was significantly decreased in group H (*p* = 0.036), and there was also a tendency of decrease in group N.

Overall, these results suggested that MCL reduced the speed of clot formation and clot strength in both groups N and H, which is likely due to a reduction in the platelet counts.

#### Effect of hypothermia on MCL-induced coagulopathy

Based on the MCL-induced decrease in coagulation, we next compared the changes in the SLT and ROTEM findings during the MCL in both groups to clarify whether hypothermia affects this decrease. Changes in the SLT and ROTEM findings were comparable in the two groups (Fig. 5a and b). The FIBTEM assays and calculated platelet MCE also showed no significant differences between groups H and N (Fig. 5c).

These results indicated that hypothermia had no impact on coagulation associated with the intrinsic and extrinsic pathways, fibrinogen, or platelets.

## Discussion

The main finding of the present study is that hypothermia does not cause irreversible loss of coagulation in vitro. In our hypothermic storage study, CK, CRT, CFF, and platelet mapping showed no significant differences between the normothermic and hypothermic groups (Fig. 3). The MCL study further supported these results, showing no difference in the SLT and ROTEM parameters between the two groups (Fig. 5). In a clinical setting, variation in factors such as patient characteristics, surgical procedures, and preoperative medication make it quite difficult to clarify the mechanism of coagulopathy in cardiac surgery under HCA [13]. This is the first study to clarify the effects of hypothermia and CPB on blood coagulation under conditions where other clinical factors are eliminated.

In cardiac surgery, especially under HCA, coagulopathy is a major problem leading to increased complications and mortality. Both CPB and hypothermia are believed to be the main causes of coagulopathy. Currently, many surgeons use higher temperatures during HCA, more than 30 °C in some cases, to reduce the risk of coagulopathy [7, 14]. While several studies have reported no increase in complications or successful reduction in the risk of bleeding [15], the safety of higher temperature settings has not been validated. On the other hand, while some studies have pointed to the risk of increased complications in the brain, spinal cord, and visceral organs associated with high temperatures during HCA [8, 16, 17], others found no reduction in bleeding or transfusion [2, 18, 19].

Hypothermia has been reported to impair blood coagulation in vitro, based on TEG and ROTEM analyses [20, 21]. These studies analyzed blood samples at hypothermic temperatures without a rewarming step. In the clinical setting, however, patients who undergo cardiac surgery under HCA are rewarmed after CPB. Therefore, the reduced coagulation under hypothermia is not an issue if it can be reversed by rewarming, and it would be meaningful to assess the status of coagulopathy after rewarming. We demonstrated that the coagulation of rewarmed blood after being exposed to hypothermia is comparable with that of the blood maintained at normothermic temperature, suggesting that the coagulation can recover after rewarming, at least in vitro.

As it is well known that CPB causes coagulopathy, we reproduced the reduction in coagulation after MCL. There are some reports showing that a decrease in fibrinogen during CPB causes coagulopathy, and that monitoring of FIBTEM MCF and administration of fibrinogen concentrate are beneficial [22, 23]. However, several randomized studies have failed to prove the efficacy of using fibrinogen concentrate in cardiac surgery with CPB [24, 25]. Our ROTEM results showed that MCF and MCE were decreased in both the INTEM and EXTEM assays, but not in the FIBTEM assay, indicating that the contribution of fibrinogen to the MCL-induced reduction of clot strength was limited. This finding could explain how clinical studies failed to observe a benefit with fibrinogen concentrate use.

We previously reported that platelet MCE is a significant parameter for monitoring coagulopathy, especially during HCA [9]. Our findings also show a decrease in platelet MCE after MCL, and no changes in the FIBTEM assay (Fig. 4c). Consistent with these data, the platelet counts were also significantly reduced after MCL (Fig. 4a). Our findings provide further evidence that the contribution of platelets to clot strength is more significant than that of fibrinogen.

## Limitations

There are two major limitations to the present study. First, this study employed blood samples provide by healthy volunteers and employed an in vitro system to eliminate clinical biases. Although this experimental design allowed us to clarify the pure effect of temperature on blood, the effects of the body’s response and the patients’ clinical background were not accounted for. Therefore, further in vivo and clinical studies will be needed to fully understand the optimal temperatures for safe HCA. Second, it is unclear whether the MCL setting was optimal for testing hypothermia- and CPB-induced coagulopathy because few previous studies have addressed this issue, and there is no standardized method. Therefore, we believe that the present study provides a valuable platform for other researchers to refer to and further optimize their MCL settings.

## Conclusion

In conclusion, our findings demonstrate that hypothermia does not cause an irreversible reduction in blood coagulation, although CPB can cause coagulopathy. Therefore, a higher temperature setting during HCA is not necessarily beneficial for preventing coagulopathy. It could instead increase the risk of organ complications within acceptable CPB times. However, further in vivo studies are needed to understand how the body’s response to hypothermia contributes to coagulopathy during HCA.
